# Cost and performance data for residential buildings fitted with GSHP systems in Melbourne Australia

**DOI:** 10.1016/j.dib.2017.03.028

**Published:** 2017-03-18

**Authors:** Qi Lu, Guillermo A. Narsilio, Gregorius Riyan Aditya, Ian W. Johnston

**Affiliations:** Department of Infrastructure Engineering, The University of Melbourne, Australia

## Abstract

The data reported in this article presents actual installation costs and performance data for a selection of residential Ground Source Heat Pump (GSHP) systems in Melbourne, Australia. The installation cost data includes five main cost components: ground loop installation, head pipe installation, heat pump, mechanical room installation, and fittings. The performance data presented here includes timestamp, air temperature and thermal loading. A more comprehensive analysis of this data may be obtained from the article entitled “Economic analysis of vertical ground source heat pump systems in Melbourne” (Q. Lu, G.A. Narsilio, G.R. Aditya, I.W. Johnston, 2017) [1].

**Specifications Table**

**Table:** 

Subject area	*Civil engineering*
More specific subject area	*Shallow geothermal; ground source heat pump system*
Type of data	*Table*
How data was acquired	*Flow meter: MT-KD-P multi-jet water meter by Bar Meters*
	*Temperature sensor: NTC-NG6K by Dixell*
	*Current transformer: CT132 by Hobut*
	*Power meter: Ipro Genius IPG100D by Dixell Emerson*
	*Data logger: XWeb 500D by Dixell Emerson*
Data format	*Analyzed*
Experimental factors	*No pretreatment of samples was performed*
Experimental features	*Cost data gathered during the installation*
	*Performance data gathered during the operation of the heat pumps*
Data source location	*Melbourne, Australia*
Data accessibility	*Data is with this article*

**Value of the data**

• The detailed thermal loading data provided for three residential buildings in Melbourne would give designers a better understanding of household׳s energy demand.
• The relationship between the thermal loading and the ambient air temperature would be extrapolated and examined using the dataset. Such relationship would then be used to compare with the Bin Method, which is commonly used to design the residential thermal loading.
• The building balance point temperature under Melbourne conditions would be better understood, and of use for other similar temperate climate locations and construction envelops around the world.
• The detailed cost breakdown of a GSHP system were presented, which would enable a cost analysis and comparisons with other conventional heating/cooling systems.

## 1 Data

**Fig. 1:**
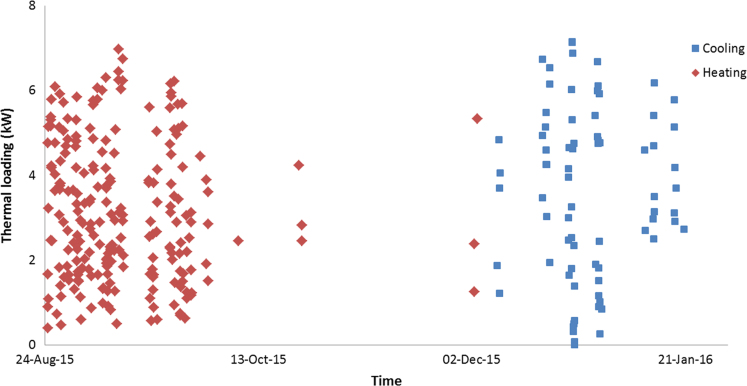
Hourly thermal loading of property 1.

**Fig. 2:**
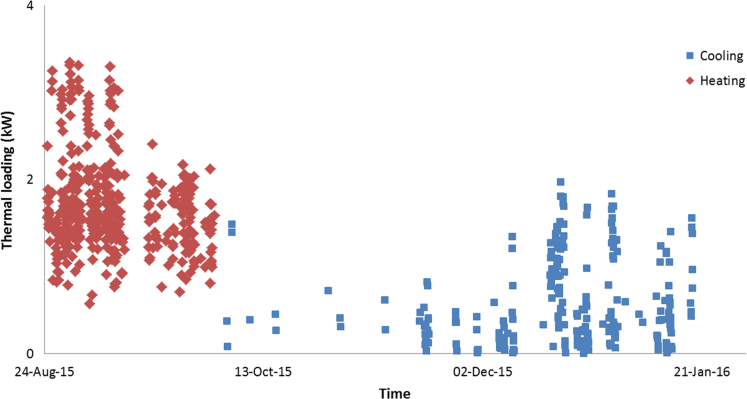
Hourly thermal loading of property 2.

**Fig. 3:**
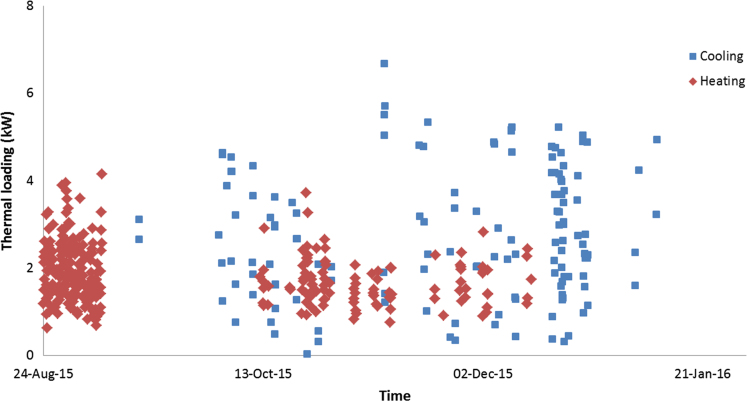
Hourly thermal loading of property 3.

The data in this data article has been gathered under a ‘Direct geothermal energy research and demonstration project’ run under the Sustainable Energy Pilot Demonstration (SEPD) program funded by the state government of Victoria, Australia. Under this project, around 20 buildings were selected for the installation of vertical GSHP systems as less expensive horizontal systems were not an option due to land space limitations. Most of these buildings are typical residential properties of 130–160 m^2^ with 2–3 bedrooms, and their thermal energy consumptions were monitored and recorded using various instruments as described in the table above. Fig. 1, Fig. 2, Fig. 3 illustrate the hourly thermal loading of three monitored residential properties. Table 1 shows the capital cost of the installed Ground Heat Exchanger (GHE).

## 2 Experimental design, materials and methods

The data logger installed in each property under the SEPD program records a range of parameters in a time interval of five minutes, including electricity consumption of the heat pump, flow rate of circulating fluid through the ground heat exchanger, ambient air temperature, entering water temperature from the ground to the heat pump, and leaving water temperature from the heat pump to the ground.

The collected installation cost data was categorized into five main components. The ground loop installation includes drilling of boreholes, grouting, purchasing and installing geothermal loop. The head pipe installation includes digging trenches, connecting geothermal loops to head pipes and installation of head pipes. The mechanical room installation includes the installation of the circulation pump, expansion tank and the connection of the header pipes to the heat pump unit. The fittings cost includes the purchase and installation of all connectors and fittings. More details can be found in [1].

## Figures and Tables

**Table 1:** Ground loop installation cost (in Australian dollars).

Drilling depth	Total (per m)	Drilling alone (per m)	Grouting (per m)	Geoloop (per m)	Labor (per m)
2× 60 m double loop GHE	$131.22	$71.50	$15.95	$14.63	$29.14
2× 60 m double loop GHE	$136.31	$55.00	$22.00	$19.44	$39.87
2× 65 m double loop vertical GHE	$96.77	$55.00	$22.00	$-a	$19.77
2× 50 m double loop vertical GHE	$104.80	$55.00	$22.00	$-a	$27.80
2× 55 m single loop GHE	$124.38	$66.00	$20.08	$15.60	$22.70
2× 55 m single loop GHE	$129.89	$55.00	$22.00	$21.20	$31.69
2× 60 m double loop GHE	$115.23	$60.50	$20.21	$-a	$34.51
2× 60 m double loop GHE	$118.25	$55.00	$22.00	$-a	$41.25
2× 60 m double loop GHE	$131.23	$71.50	$16.83	$-a	$42.90

a When the Geoloop component has zero cost, it means that the total cost (per m) does not include Geoloop.

## References

[1] Q. Lu, G.A. Narsilio, G.R. Aditya, I.W. Johnston, Economic analysis of vertical ground source heat pump systems in Melbourne, Energy 125 (2017) 107-117, 10.1016/j.energy.2017.02.082

